# Harnessing NKT cells for vaccination

**DOI:** 10.1093/oxfimm/iqab013

**Published:** 2021-06-19

**Authors:** Olivia K Burn, Theresa E Pankhurst, Gavin F Painter, Lisa M Connor, Ian F Hermans

**Affiliations:** 1 Malaghan Institute of Medical Research, PO Box 7060, Wellington 6042, New Zealand; 2 The School of Biological Sciences, Victoria University of Wellington, PO Box 600, Wellington 6140, New Zealand; 3 The Ferrier Research Institute, Victoria University of Wellington, PO Box 33436, Petone 5046, New Zealand; 4 Maurice Wilkins Centre for Molecular Biodiscovery, The University of Auckland, Private Bag 92019, Auckland, New Zealand

**Keywords:** NKT, vaccine, glycolipids, adaptive immunity, innate-like T cells

## Abstract

Natural killer T (NKT) cells are innate-like T cells capable of enhancing both innate and adaptive immune responses. When NKT cells are stimulated in close temporal association with co-administered antigens, strong antigen-specific immune responses can be induced, prompting the study of NKT cell agonists as novel immune adjuvants. This activity has been attributed to the capacity of activated NKT cells to act as universal helper cells, with the ability to provide molecular signals to dendritic cells and B cells that facilitate T cell and antibody responses, respectively. These signals can override the requirement for conventional CD4^+^ T cell help, so that vaccines can be designed without need to consider CD4^+^ T cell repertoire and major histocompatibility complex Class II diversity. Animal studies have highlighted some drawbacks of the approach, namely, concerns around induction of NKT cell hyporesponsiveness, which may limit vaccine boosting, and potential for toxicity. Here we highlight studies that suggest these obstacles can be overcome by targeted delivery *in vivo*. We also feature new studies that suggest activating NKT cells can help encourage differentiation of T cells into tissue-resident memory cells that play an important role in prophylaxis against infection, and may be required in cancer therapy.

## THE NECESSITY OF NEW ADJUVANTS FOR VACCINATION

While there are highly effective vaccines against a variety of infectious agents, there are still many infectious and chronic diseases for which vaccines are not available, or where the available vaccines are suboptimal in terms of immunogenicity, safety and cost. Furthermore, although the discovery of subunit vaccines has dramatically improved safety profiles, purified antigenic components are generally poorly immunogenic, making it critical that they are co-administered with adjuvants. Adjuvants are defined as compounds that can enhance the magnitude, function or breadth of an induced immune response and enable dose sparing [1]. Currently, there are only a handful of adjuvants approved for human use, with aluminium salts (alum) the most commonly used. Alum was originally thought to enhance immune responses solely by sequestering antigen as depots at the injection site, although subsequent studies suggest a more complicated (if not fully understood) mechanism [2, 3] involving an additional pro-inflammatory response that ultimately stimulates recruitment and activation of dendritic cells (DCs) [4]. Other approved adjuvants, such as oil-in-water emulsions using squalene, DL-a-tocopherol (vitamin E), or lipids from the *Salmonella minnesota* bacterium or *Quillaja saponaria* tree, likely involve variations on the same themes of enhanced availability of antigen, and immunostimulation leading to DC activation [5]. More recently approved adjuvants have focused on engaging pattern recognition receptors (PRRs) expressed by DCs. These include the toll-like receptor (TLR)-4 agonist monophosphoryl lipid A (MPLA), the TLR-2 agonist Pam3Cys, the TLR-7 and -8 agonist imidazoquinoline resiquimod (R-848) and the TLR-9 agonist cytosine–guanosine (CpG) [6]. The recently approved mRNA-based vaccines for protection from Severe acute respiratory syndrome coronavirus 2 (SARS-CoV-2) infection [7, 8] rely on the immunostimulatory properties of the nucleic acid itself as in-built adjuvants, acting via TLR7/8 and -9, highlighting a key advantage of this exciting new vaccine platform.

This review focuses on the adjuvant activities of agonists for type I natural killer T (NKT) cells, a population of ‘innate-like’ T cells that express stereotyped T cell receptors (TCRs) (Vα14Jα18 paired with Vβ8, Vβ7 or Vβ2 in mice and Vα24Jα18/Vβ11 in humans) that recognize defined non-peptide ligands (typically glycolipids) presented by the non-polymorphic major histocompatibility complex (MHC) Class I-like CD1 family member CD1d. These agonists act as adjuvants via a unique mechanism involving the recruitment of NKT cells to function as ‘universal helper’ T cells to enhance cellular and humoral immune responses against co-administered antigens. It is becoming increasingly clear that NKT cell responses can be manipulated via changes in the structure of the NKT cell agonists (as we recently reviewed with regard to cancer [9]). Here we emphasize the unique features of ‘NKT cell adjuvanted’ vaccines that enable them to enhance adaptive responses to many different diseases, and highlight how improvements in modes of delivery can also enhance the efficacy and safety of NKT cell help.

## GENERATING ADAPTIVE IMMUNE RESPONSES

The generation of adaptive immune responses to vaccination requires uptake of antigenic material from the injected tissues or circulation, and presentation of the acquired material to T cells in the form of antigenic peptide bound to MHC molecules. Within a host’s diverse repertoire of T cells, where each cell expresses a uniquely recombined TCR for antigen recognition, there is a high likelihood that a T cell will recognize a given peptide/MHC complex. To induce clonal expansion of these cells, exogenous antigens delivered by vaccination must be acquired by DCs and processed within the vacuolar compartments into peptides for presentation via MHC. Vacuolar processing typically leads to presentation of peptides on MHC Class II molecules to CD4^+^ T cells, although some processing can lead to cross-presentation via MHC Class I molecules to CD8^+^ T cells. Such cross-presentation is also achieved through antigen escape from the phagolysosome, with processing in the cytosol providing peptides that are ultimately exported to the surface by MHC Class I molecules [10]. In both humans and mice, a subset of conventional DCs, XCR1^+^ DCs (termed cDC1s), has a heightened capacity for cross-presentation, and are therefore critical in the initiation of vaccine-induced CD8^+^ T cell responses.

The ability of DCs to stimulate proliferation and differentiation of T cells requires their activation, which in the case of infection can be triggered by the infectious microbes themselves, through recognition of pathogen-associated molecular patterns or recognition of damage-associated molecular patterns released by damaged or dying cells in infected tissues. Recognition is via innate PRRs expressed on the DC surface or within phagolysosomal compartments where they come into contact with acquired microbial products. As already noted, compounds that stimulate via PRRs have become useful vaccine adjuvants, notably agonists for the TLRs. In response to PRR stimulation, DCs upregulate peptide presentation on MHC and express co-stimulatory molecules, such as CD80 and CD86, as well as increased production of proinflammatory cytokines interleukin (IL)-12 and type I interferons (IFNs) [11, 12], enhancing the effector T cell response against the presented peptide [13]. Although pattern recognition alone is sufficient to enable DCs to induce T cell proliferation, it is when this is accompanied by T cell ‘help’ in the form of direct molecular interactions and cytokines that long-lived functional T cell responses are induced, notably the differentiation of CD8^+^ T cells into cytotoxic cells [14, 15]. The help provided by CD4^+^ T cells is primarily mediated through CD40L/CD40 interactions [16–18], leading to further increases in the expression of MHC molecules and co-stimulatory molecules, and significantly enhanced production of key cytokines, such as IL-12p70. For the induction of CD8^+^ T cell responses, antigen-specific CD4^+^ T helper cells and CD8^+^ T cells must be brought together by an antigen-loaded DC that displays peptide epitopes via MHC molecules to both cell types. The three cells need not meet simultaneously, but rather the helper cell can engage the DC first to ‘licence’ it to then be capable of stimulating full differentiation of CD8^+^ T cells [17]. Note that it is a similar function, again mediated via CD40L/CD40 interactions, which enable T follicular helper (T_FH_) cells to trigger B cells to produce antibodies [19]. Thus, in the context of vaccination strategies, it is critical to provide or mimic the signals provided by pattern recognition and T cell help to induce cellular and humoral adaptive immune responses.

Given the high degree of inter-individual MHC polymorphism, inducing the appropriate quality of T cell help may not be assured in all individuals by vaccination. Naïve CD4^+^ T cells specific for a given peptide within an individual’s repertoire can be rare [20], and it is known that TCR diversity declines with age [21, 22], potentially leading to gaps where specific antigens may not be recognized [23, 24]. Similarly, in designing subunit vaccines, selected antigens may simply have few suitable helper epitopes, despite being a good target for other immune effectors. This issue is even more of a problem when peptide-based vaccines are used; these should contain MHC Class II-binding epitopes to elicit CD4^+^ T cell help, but currently selecting sequences based on MHC Class II-binding algorithms is imprecise.

## NKT CELLS CAN PROVIDE T CELL HELP

Studies conducted on NKT cells showed that they expressed CD40L and could induce activation of DCs *in vitro*, suggesting they could be useful helper T cells [25]. A key advantage to exploiting NKT cells in this role is their high frequency. In mice, NKT cells are abundant in liver (10–30% of liver lymphocytes) and spleen (0.5–3%), with lower frequencies in thymus, blood, bone marrow (0.4–8%) and lymph nodes (0.1–0.2%) [26]. In humans, there is a substantial inter-individual variability, with total frequency considerably lower than mice, with additional differences in tissue distribution; nonetheless, frequency of NKT cells in lymphoid tissues is high relative to antigen-specific conventional CD4^+^ T cells. Because the TCR structure of NKT cells is largely invariant, and polymorphism in their restriction element, CD1d, is extremely low; it was anticipated that their helper function could be readily triggered with the same specific agonists in all individuals. In fact, CD1d structure is conserved among many mammalian species [27], conveniently meaning it is possible to conduct pre-clinical studies of potentially clinically relevant vaccines that exploit NKT cells in animal models. The most commonly used agonist has been the glycolipid α-galactosylceramide (α-GalCer), which was developed through structure–activity relationship studies on an extract of the Okinawan marine sponge *Agelas mauritianus* with anti-tumour activities in mice [28–31]; the anti-tumour activity was subsequently attributed to cytokine production through the specific activation of NKT cells [32, 33]. Another key difference between NKT cells and conventional T cells is the kinetics of response to stimulation, with NKT cells exhibiting functional activity within hours of engagement, rather than several days for conventional T cells [34]. In fact, NKT cells have stores of preformed cytokine mRNA enabling rapid release of large quantities upon TCR engagement [35]. As a consequence, NKT cells represent a numerically rich population of cells that bridge innate and adaptive immunity that can be rapidly mobilized with specific antigens. In early *in vivo* studies, injection of α-GalCer into mice was shown to increase expression of activation markers on T and B cells [36], and antigen-specific CD4^+^ T cell responses were induced when α-GalCer was combined with chicken ovalbumin (OVA) protein [37]. Later it was shown that CD8^+^ T cell responses could be raised against malaria antigens [38], and several studies followed that reported α-GalCer-enhanced CD4^+^ and CD8^+^ T cell responses to protein and peptide antigens, including tumour antigens [39–41]. As expected, the induced T cell responses involved CD40 signalling, with activation of DCs also involving tumour necrosis factor and type I and II IFNs [39, 40, 42]. Although the helper activity was similar to conventional CD4^+^ T cell help, differences in the chemokine profile released by DCs after interaction with NKT cells were noted, with release of CCL17 being prominent in attracting naïve CCR4^+^ CD8^+^ T cells [43]. Another significant factor was the release of significant quantities of IL-12p70 [44], an important pro-inflammatory cytokine that helps drive T cell differentiation. This was largely attributed to CD8α^+^ DCs in mice [45, 46], now considered to be cDC1s—the same cell-type shown to have a high propensity for cross-presentation and priming of CD8^+^ T cell responses. Two features of these early studies are of importance with respect to exploiting NKT cells as cellular adjuvants in vaccination platforms. The first is that the powerful adaptive responses induced were observed in the absence of providing any form of pattern recognition, meaning that agonists for NKT cells could be used in vaccines as stand-alone agents. This is not to say that pattern recognition cannot contribute to the response. In fact, it was found that the helper function of NKT cells cooperated effectively with TLR agonism, resulting in even stronger adaptive immune responses, although this was associated with considerably enhanced cytokine release that would have to be managed in a vaccine setting [47, 48]. The second feature of relevance to vaccines was that responses could be induced in mice deficient of conventional CD4^+^ T cells, meaning that NKT cells can compensate for lack of CD4^+^ T cell help, and vaccines can be designed without consideration for the inclusion of helper epitopes [41, 49].

## INDUCING RESPONSES WITH FREE NKT AGONIST AND ANTIGEN

The most straightforward strategy to utilize NKT cell agonists as immune adjuvants is to simply co-administer the agonist with the antigens of interest. Some of the earliest studies were conducted with malaria sporozoites as the source of antigens [38], resulting in a significant T cell-mediated immunity, which were followed by studies using model antigens [40], pathogen-derived antigens [50, 51] and tumour-associated antigens [47, 52]. Some of the most extensive work has been conducted on improving the efficacy of inactivated influenza virus vaccines (reviewed in Driver *et al.* [53]). Co-administration of NKT cell agonists with inactivated influenza A was shown to enhance both cellular [54–56] and humoral responses [50, 57], resulting in improved protection against virus challenge in mice. One study showed that the inclusion of α-GalCer had the paradoxical effect of limiting the immediate CD8^+^ T cell response, but promoted the survival of long-lived memory populations capable of boosting protection against heterologous influenza A virus challenge [56]. Others, using an intranasal route of delivery, also reported enhanced cross-protection against heterologous and heterosubtypic viral infections [58], which they attributed to the additional impact of NKT cell accumulation in the lungs, and increased IgA production. Studies have also been conducted in pigs, where co-administration of α-GalCer with inactivated swine influenza variants OH07 or CA04 induced systemic and mucosal influenza-specific antibodies in upper and lower respiratory tracts and reduced pulmonary viral load after virus challenge [59–61]. In moving forward to studies in non-human primates, an initial study using α-GalCer with inactivated influenza virus in macaques showed that NKT cells were modulated by the treatment, but with no impact on antiviral immunity [62]. However, while there was a close temporal relationship between delivery of the glycolipid and inactivated virus, the agents were not co-delivered by the same route, so may not have accessed the same antigen-presenting cells (APCs) to enable the licencing events to occur. It is important to note that activated NKT cells are useful effectors themselves, and can limit virus infection via other mechanisms, including reducing suppressive capacity of myeloid suppressor cells [63], activating NK cells [64], directly lysing infected cells [65] and producing cytokines that protect the lung epithelium from damage [66].

A key factor to consider in the use of NKT cell agonists as immune adjuvants in humans is the difference in NKT cell frequency between the commonly used rodent models and humans, where peripheral numbers are >30-fold lower [67–69]. It is therefore notable that in a cohort of pigs with mixed genetic background, where there was a range of NKT cell frequencies similar to humans, co-administration of α-GalCer with model antigen hen egg lysozyme improved antigen-specific antibody production [70]. In fact, NKT cell frequency in peripheral blood measured prior to treatment was a poor predictor of how individual animals responded. These results were also encouraging as pigs and human NKT cells share key phenotypic characteristics, including a large double negative NKT cell population and a subset of CD8^+^ NKT cells, which are absent in mice. In terms of clinical development of the adjuvant concept, ABX196, a variant of α-GalCer with a galactosyl 6-deoxy-6-N-acyl modification that showed more potent agonistic activity in mice, has been assessed in combination with hepatitis B virus surface antigen (HBsAg) in healthy volunteers. This resulted in activation of NKT cells in the majority of participants, with the response again not predicted by prior NKT cell frequency in peripheral blood. Importantly, antibody HBsAg-specific antibody titres reached protective levels in 79% of the patient cohort, as opposed to 50% in participants that received a commercial hepatitis B vaccine [71].

This same study highlighted another important consideration in vaccine development; the risk of associated toxicity. While it had been shown that mice exhibit transient mild hepatoxicity in response to systemically administered α-GalCer [72–74], this had not been seen when α-GalCer was administered in a dose-escalation study to patients with solid tumours, despite a wide range of doses used (50–4800 µg/m^2^) and evidence of NKT cell activation [75]. Nonetheless, when ABX196 was used in the above trial, 3 of the 44 patients developed elevated ALT levels associated with transient moderate alterations in liver biology that justified their withdrawal from the study. The authors reasoned that it is necessary to limit access of the glycolipid to the liver, based on evidence that α-GalCer can support T cell responses with significantly reduced hepatoxicity in mice deficient in fatty acid amide hydrolase where liver transport of the glycolipid is compromised [76]. In a post-clinical evaluation, they showed evidence that this can be achieved by formulation of ABX196 into an emulsion containing oil, solvent and surfactants as opposed to the liposomal formulation used in the clinical study. In another clinical study conducted with α-GalCer alone in patients with hepatitis B virus, 4 of 27 patients discontinued treatment due to rigours [77], highlighting the need to also consider the disease status of individuals undergoing treatment (interestingly, hepatitis C patients were not similarly affected).

Studies in mice have also raised another potential limitation to using NKT cells agonists as adjuvants; the induction of a period of NKT cell hyporesponsiveness, sometimes referred to as anergy, where the cells produce less IFN-γ upon re-stimulation, but do produce IL-4, IL-13 and even the suppressive cytokine IL-10 [78]. This period of anergy may limit the helper function of NKT cells, suggesting NKT cell agonists could be poor adjuvants in prime-boost vaccination schedules. Some studies suggest that anergy is due to presentation of NKT cell agonists by non-professional APCs, and can be improved by strategies to specifically target appropriate APCs. Also, a number of structure activity relationship studies have been conducted on NKT cell agonists to improve cytokine profile and reduce anergy, potentially as a consequence of altered biodistribution and rate and route of presentation (reviewed elsewhere [9, 79–81]). Overall, although there is an evidence that inducing NKT cell help with injected free agonists can improve adaptive immune responses, development of the concept in humans has been hampered by issues associated with effective and safe delivery. Progress towards clinical translation will require further development where improvements in efficacy must be evaluated against risk of toxicity.

## α-GALCER-LOADED ANTIGEN-EXPRESSING CELLS

One way to avoid the issues associated with injecting free agonists is to load the agonists onto injected antigen-bearing DCs, which were shown to be an effective way to elicit strong CD8^+^ T cell responses in animal studies [41, 82, 83]. Furthermore, an early study showed that when α-GalCer was pulsed onto DCs, rather than administered as a free agent, minimal anergy of the NKT cell population was observed [84]. Although it was initially thought that the injected cells were the recipients of NKT cell-mediated licencing, studies in mice highlighted a role for host cDC1 cells in both IL-12p70 production and enhancement of the induced CD8^+^ T cell response [85–88]. These results imply transfer of antigen and α-GalCer to resident cells, which are then licenced by NKT cells, although the mechanism remains unclear. The fact that injection of CD1d-deficient DCs loaded with antigen and α-GalCer could also induce strong T cell responses reinforced this concept [87]. Our own clinical studies may also be of relevance. We assessed T cell responses in a Phase I clinical trial of DCs co-pulsed with peptide and α-GalCer in high-risk melanoma patients [89], and more recently completed a randomized Phase II study where patients were assigned to receive peptide-loaded DCs with or without α-GalCer (ACTRN12612001101875). While strong immune responses to the NY-ESO-1 peptides loaded onto the DCs were observed in the vast majority of patients, no significant enhancement in the level of T cell response was seen when α-GalCer was included in the vaccine. Although there were other confounding factors, including an unexpectedly low NKT cell response, a possible interpretation is that transfer of antigen and/or agonist to resident DCs was inefficient or not achieved, which may reflect the antigens used (long peptides) and potentially the formulations used for loading the compounds onto the DCs. If transfer is indeed required, a consequence is that the efficacy of the transfer process is of more importance to vaccine outcome than the cell type used as the vector for the glycolipid and antigen. In fact, preclinical studies have shown that irradiated tumour cells pulsed with glycolipid [90–92], or antigen mRNA-transfected fibroblasts loaded with α-GalCer [93] can generate T cell responses that benefit from NKT cell licencing. Nonetheless, the type of injected cell may still be of relevance, as some cells may have a greater capacity for antigen transfer than others. In this regard, it was recently shown that monocytes are more efficient at the task of transferring peptide antigens than monocyte-derived DCs [94].

## NANOSCALE DELIVERY SYSTEMS

If endogenous DCs are critical to the cellular adjuvant activities of NKT cells, it would be more efficient to design vaccines that can target these cells directly, especially as ex vivo expansion and loading of DCs is time-consuming and costly. Several NKT cell-activating vaccines have therefore made use of nanovectors as delivery systems to ensure co-delivery of antigen and adjuvant to the same cell *in vivo* (Table 1).

**Table 1:** Nanoscale glycolipid-peptide antigen vaccines

Nanovector	Adjuvant	Antigen	Target molecule and NKT cell response	Cellular/humoral immune response and disease outcome	Reference
PLGA nanoparticle	α-GalCer	OVA	DEC205 to target splenic and lymph node resident CD8α^+^ DCs Activated NKT cells *in vivo* with less anergy upon restimulation	Increased effector and memory CD8^+^ T cell response Increased OVA-specific IgG titre Prophylactic and therapeutic vaccination delayed B16.OVA tumour growth	[46]
PLGA nanoparticle	α-GalCer	OVA	Induced IL-2 production by hybridoma NKT cell line *in vitro*	Prophylactic and therapeutic vaccination delayed B16.OVA tumour growth	[95]
PLGA nanoparticle	IMM60	NY-ESO-1	Induced IL-2 production by hybridoma NKT cell line *in vitro*	Increased antigen-specific T cell responses and antibody levels	[96]
PLGA nanoparticle	IMM60	OVA	I.V. administration activated Th1 type NKT cells I.N. or S.C. administration activated Th17 NKT cells	Therapeutic vaccination delayed B16.OVA and HPV-expressing TC-1 tumour growth	[97]
PLGA nanoparticle	α-GalCer, MPLA & CpG ODN	Melan-A and gp100	Increased percentage of NKT cells in the tumour	Therapeutic vaccination delayed B16.F10 tumour growth	[98]
PLGA nanoparticle	α-GalCer	Melan A	Activated and expanded human NKT cells in HIS-CD8/NKT mice	Increased frequency of Melan A^+^ CD8^+^ T cells	[99]
Gold nanoparticle	α-GalCer	MUC1	Splenocytes released more IFN-γ and IL-4, most likely from activated NKT cells	Increased MUC1-specific cytotoxicity Therapeutic vaccination delayed B16.MUC1 tumour growth	[100]
LNPs	α-GalCer	Ova mRNA	Increased frequency of NKT cells in spleen and liver	7× more OVA-specific cytotoxic T cells in tumour Rejected 40% of EG7-OVA tumour in therapeutic setting Synergized with CPI	[101]
LNPs	α-GalCer	TRP2 mRNA	Activated NKT cells	Increased frequency of TRP2^+^ CD8^+^ T cells in periphery and tumour Therapeutic vaccination delayed B16.F10 tumour growth	[102]
Liposomes	α-GalCer	gp100	Lewis-Y to target DCs and Langerhans cells Increased NKT cell activation *in vitro*	Activated gp100^+^ CD8^+^ T cells *in vitro*	[103]
Liposomes	α-GalCer	OVA	GM3 to target CD169^+^ macrophages Activated NKT cells	Increased frequency of OVA^+^ CD8^+^ T cells and production of IFN-γ by CD8^+^ T cells	[104]
Liposomes	α-GalCer	Tn antigen		Increased IgG antibody responses	[105]
Liposomes	α-GalCer	MUC-1		Increased IgG antibody responses	[106]
Cationic liposomes	α-GalCer	TRP2	Activated NKT cells *in vitro*	Prophylactic vaccination delayed B16.F10luc2 tumour growth	[107]
VLPs	α-GalCer	gp33	Activated splenic NKT cells to produce IFN-γ and IL-4.	Increased frequency of gp33^+^ T cells Prophylactic vaccination delayed B16.gp33 tumour growth	[108]
Nanoemulsion	α-GalCer	OVA HPV.E7	Clec9a-targeted Activated NKT cells	Enhanced antigen-specific CD8^+^ T cell response Therapeutic vaccination delayed TC-1 tumour growth	[109]
Filamentous bacteriophage *fd*	α-GalCer	OVA	Activated NKT cells	Increased OVA^+^ CD8^+^ T cells Therapeutic vaccination delayed B16.OVA tumour growth	[110]

Nanovectors (<1 μM) comprise a range of particulate systems including nanogels, nanoemulsions, liposomes, carbon nanotubes, virus-like particles (VLPs), silica microspheres, metallic nanoparticles and polymeric nanoparticles (which include nanospheres and nanocapsules). Nanovectors display several desirable properties including capacity to incorporate antigen at high density, the ability to deliver the antigenic payloads to the MHC Class I pathway and permit slow extended release of the payload, which may benefit the initiation of immune responses [111–114]. The physical and chemical properties of nanovectors influence size, shape and surface charge, with these factors in turn determining the location of drug delivery and DC uptake *in vivo* [115]. The advantages and disadvantages of these nanovectors have been extensively reviewed elsewhere (see Ghinnagow *et al.* [116]).

Several polymers have been used to produce nanovectors, with poly(lactic-co-glycolic) acid (PLGA) showing the most success to date resulting in FDA approval for PLGA in medical applications [117]. Following the demonstration that PLGA nanovectors containing NKT cell agonists can be internalized by DCs to promote NKT cell activation [118, 119], nanovector vaccines were evaluated where both NKT cell agonist and peptide antigens were included. These nanovector vaccines, containing α-GalCer or IMM60 (a novel class of NKT cell agonist with a non-carbohydrate structure—threitol—coupled to the ceramide moiety), were consistently better at inducing anti-tumour responses than co-administration of free or individually entrapped agonist and antigen [95, 96]. Another report showed that similar vaccines induced prolonged immune responses when compared with injection of the separated components [120]. This was supported by a recent study that showed greater B cell activation, with higher-titre antigen-specific IgM and IgG responses that were maintained long term [121]. Significantly, the vaccines activated NKT cells without inducing anergy.

A recent study explored the use of gold-based nanoparticles containing a six-substituted α-GalCer analogue and the tumour-associated antigen MUC-1 [100]. Both agonist and antigen were attached to the gold particles via amide bond formation with a reactive N-hydroxysuccinimide ester that had been previously deposited on the surface via Au-thiolate bonding. These nanoparticles were found to induce both cellular immune and humoral responses, and induce anti-tumour activity. Interestingly, smaller particles (∼60 nm) induced stronger responses, which may reflect a more efficient passage from interstitium at the injection site to lymphatic capillaries for transfer to lymph nodes. Also, the smaller size meant less α-GalCer per particle, which may have driven a qualitatively different signal to NKT cells, potentially with less anergy in the repeated dosing regimen used, although this was not explicitly examined.

VLPs have also been shown to be effective vectors for co-delivery, with the added advantage that recombinant technology can be used to introduce an antigen of interest. This strategy was used to generate recombinant VLPs incorporating an epitope from lymphocytic choriomeningitis virus glycoprotein (gp33) at the N-terminus of the capsid protein of rabbit haemorrhagic disease virus. The capacity for these VLPs to bind carbohydrates enabled α-GalCer to be attached via its galactose moiety. These particles induced strong CD8^+^ T cell responses and effective anti-tumour responses against gp33-expressing B16 melanoma [108]. Filamentous bacteriophages are often referred to as natural nanocarriers, as a result of their nano-size and capacity to cross blood vessels while expressing large amounts of recombinant protein antigen. Filamentous bacteriophages expressing OVA antigen and conjugated to α-GalCer enhanced OVA-specific CD8^+^ T cell responses when administered *in vivo*, and, in the B16.OVA model, intra-tumoural administration delayed tumour outgrowth and improved survival [110].

Lipo-nanoparticles (LNPs) composed of cationic lipids combined with other neutral phospholipid molecules and sterols, such as cholesterol, have been developed as nucleic acid carriers, and are key to the success of mRNA vaccines. Co-delivery of unmodified OVA-encoding mRNA with α-GalCer in an LNP platform resulted in anti-tumour activity with high levels of tumour-infiltrating T cells, fewer tumour suppressor cells and greater anti-tumour response than nanoparticles expressing OVA mRNA only, with 40% of established EG7-OVA tumours completely rejected [101]. These α-GalCer-adjuvanted mRNA vaccines also synergized with anti-PD-L1, an immune checkpoint inhibitor (CPI), to limit tumour outgrowth reduction and significantly prolonged median survival. Evidence was provided to show that DCs were activated *in vivo* suggesting the mRNA-encoded antigen was translated and presented in the context of NKT cell-mediated licencing. In another study, α-GalCer was included with mRNA for the antigen tyrosinase-related protein 2 (TRP2) in a lipopolyplex vector, where the mRNA was complexed with a poly-(β-amino ester) polymer and entrapped into a lipid shell containing the glycolipid. This vaccine provided a significant therapeutic effect against the B16-F10 melanoma model [102]. Given that the delivery of mRNA has the advantage that manufacturing of antigenic subunits is not required, these studies represent a significant practical advance in translation to the clinic.

Targeting of glycolipid/antigen nanoparticles to the desired cell type can be achieved by decorating the surface with specific ligands. Incorporation of anti-CLEC9a antibodies into PLGA-based nanoparticles directed uptake by murine CD8α^+^ DCs and, as was recently demonstrated in a humanized mouse model, directed uptake to the human equivalent, CD141^+^ DCs [99, 122]. These nanoparticles induced strong peptide-specific CD8^+^ T cell responses and anti-tumour activity in a B16.F10 melanoma model, and in PBMCs from healthy donors and melanoma patients, induced proliferative CD8^+^ T cell responses against the tumour antigen Melan-A. Using a similar strategy, α-GalCer and the human papillomavirus (HPV) antigen E7 were encapsulated in an oil-in-water Clec9a-targeted nanoemulsion to induce anti-tumour responses to the HPV-expressing TC-1 tumour cell line in mice [109]. Enhanced cellular and humoural immune responses were also observed when PLGA-based nanoparticles containing α-GalCer and OVA peptide were decorated with antibodies against the endocytic C-type lectin receptor DEC205 that is expressed on splenic and lymph node resident CD8α^+^ DCs [46]. Another targeting approach has been to attach lipo-Lewis Y (LeY) to liposomes to facilitate C-type lectin-mediated uptake by monocyte-derived DCs, dermal DCs and Langerhans cells. This was shown to work on human skin-emigrated APCs in an explant model, inducing enhanced CD8^+^ T cell responses to the tumour-associated antigen gp100 *in vitro* [103]. Incorporating the ganglioside GM3 was shown to facilitate specific targeting to CD169^+^ macrophages in mice. Interestingly, while it appeared the CD169^+^ macrophages were capable of presenting α-GalCer to the NKT cells, generation of a peptide-specific CD8^+^ T cell response required endogenous cDC1 cells, suggesting antigen transfer was required [104]. A strategy to further improve immune responses to NKT cell-adjuvanted vaccines could be to design nanoparticles that provide direct stimulation of the APCs targeted. While two studies have shown glycolipid-antigen nanoparticle vaccines induce greater antibody titres against tumour-associated antigens than those induced by nanoparticle vaccines containing TLR agonists as the adjuvant [106, 123] further enhancement of the response could be achieved by incorporating both TLR and glycolipid agonists. This is highlighted by a recent study where the addition of α-GalCer to nanoparticles encapsulating multiple tumour antigens and the TLR agonists CpG and MPLA improved the anti-tumour response in a mouse model by 5-fold and enhanced the frequency of NKT, NK and CD4^+^ T cells in the tumour microenvironment [98].

A consideration in the use of nanoparticle vaccines is the route of administration, which can not only affect targeting to specific APC populations but can also influence the nature of NKT cell response induced. For example, intravenous administration of PLGA-entrapped NKT cell agonist IMM60 and OVA peptide resulted in activation of NKT cells in the liver and spleen, and was associated with a high IFN-γ response, enhanced T cell cytotoxicity and a Th-1 biased antibody profile featuring high levels of IgG2c [97]. The NKT cells involved were characterized as NKT1 cells, a phenotype defined on the basis of transcription marker expression (T‐bet^+^PLZF^low^) and propensity to make IFN-γ [124]. In comparison, administration via the subcutaneous or intranodal route resulted in drainage or retention in the regional lymph nodes, where more cells of an NKT17 phenotype (RORγt^+^PLZF^int^) are present, and this resulted in a decreased IFN-γ and cytotoxic response and reduced IgG2c. There was a significant survival advantage to intravenous administration of the PLGA nanoparticles in the therapeutic treatment of subcutaneous B16.OVA. A similar observation was made with cationic liposomes encapsulating α-GalCer and peptide antigen tyrosine-related protein 2 (TRP2), where anti-tumour responses were only observed when the liposomes were administered intravenously [107].

## GLYCOLIPID CONJUGATE VACCINES

Co-delivery of antigen and NKT cell agonist to the same APCs *in vivo* can also be facilitated by chemically conjugating the two components together. This strategy was used to raise antibody responses to the hapten 4-hydroxy-3-nitrophenylacetyl (nitrophenyl; NP) when it was conjugated via a six-carbon linker attached at the 2-hydroxyl of the galactose of α-GalCer [125], and antibodies to the capsular polysaccharide of *Streptococcus pneumoniae* [126] were generated when the polysaccharide was attached via the 6-hydroxyl of the galactose of α-GalCer (a position that was not expected to interfere significantly with CD1d-binding and NKT cell recognition) [127]. In both cases, the induction of activated NKT cells provided T cell help to B cells, although the quality of response was dictated by the nature of antigen attached (a concept discussed in more detail below). We have used a similar approach for attaching peptides to specifically induce T cell responses, conjugating either via a thiol introduced at the six-position of the galactose [128], or via an enzymatically cleavable linker to a prodrug form of α-GalCer where the acyl chain has been migrated to expose a free amino group on the sphingosine for attachment. The use of the linker encourages intracellular cleavage, with the peptide subsequently presented to T cells via MHC molecules, while the released inactive prodrug spontaneously converts to its active form to be presented via CD1d to NKT cells. Based on earlier studies showing that activated allergen-specific CD8^+^ T cells can reduce airway inflammation in animal models [129–131], potentially by killing the APCs that drive CD4^+^ T cell-mediated allergic responses [131], a vaccine of this design was first used to suppress allergic responses in mice [132]. Key to the success of the strategy was that allergen-specific CD8^+^ T cell responses could be licenced without the need for CD4^+^ T cells, thereby avoiding provoking the allergen-specific CD4^+^ T cells that cause disease. Variations on the same vaccine platform design, specifically relying on cathepsins for the enzymatic cleavage, were subsequently used to generate peptide-specific CD8^+^ T cells against influenza antigens [133], tumour antigens [134] and parasite antigens [135]. The conjugated vaccine also caused increased proliferation and differentiation of virus-derived peptide-specific CD8^+^ T cells when cultured with human PBMC, despite a low baseline frequency of NKT cells [136]. In all of the studies conducted *in vivo* and *in vitro*, the conjugates performed better than admixed unconjugated components, providing not only a dose-sparing advantage but also the possibility that weaker NKT cell agonists could be used. In fact, when peptide was conjugated to the weakly stimulatory monoacyl compound α-galactosylphytosphingosine, peptide-specific T cell responses could be induced in mice [137] without the transient hepatotoxicity seen when potent agonists like α-GalCer are typically used [73].

An alternative NKT cell conjugate vaccine design has been investigated based on the concept of bispecific T cell engagers (BiTEs) [138]. In this design, a photoactivable analogue of α-GalCer [139] was covalently conjugated to a soluble single-chain CD1d-β2 microglobulin fusion protein (mCD1d-β2m) to create covalently stabilized NKT cell-specific activators. By genetically fusing these with a single-chain antibody domain with specificity for a cell-surface expressed tumour-associated antigen, the direct cytotoxic effects of NKT cells could be targeted specifically at tumour tissue [140]. Notably, these BiTEs were found to induce less proliferation of NKT cells compared with administration of free α-GalCer, despite inducing similar levels of IFN-γ, IL-4 and IL-12p70 in the serum. This reduced proliferation also correlated with reduced anergy of the NKT cells, as NKT cells in mice primed 3 times previously with the BiTEs were just as responsive to restimulation with α-GalCer as those in mice primed with a single dose or saline control. While these BiTEs are not vaccines in the traditional sense, many changes within the tumour infiltrating leucocytes were observed, including enhanced CD8^+^ T cells specific against tumour-associated peptides and lysate, suggesting their mode of action promoted epitope spreading.

In each of the animal models of disease where T cell responses to conjugates have been evaluated, exploiting the helper function of NKT cells has been a valid strategy to improve adaptive responses. However, NKT cell function can be affected by disease, resulting in reduced or polarized cytokine production and reduced numbers [141, 142]. In fact, altered NKT cell activity can actually contribute to disease [143]. The decision to use NKT cell-adjuvanted vaccines in a disease setting, particularly in a therapeutic context, therefore has to be considered carefully.

## CONSIDERATIONS IN INDUCING HUMORAL IMMUNITY

As expected, co-administration of a protein antigen with α-GalCer enhances humoral immunity to the antigen in a CD1d-dependent and NKT cell-dependent manner (reviewed by Lang [145]). It is now known that NKT cells can provide help to support B cell responses via two mechanisms; an indirect, non-cognate mechanism involving interactions with antigen-loaded DCs that lead to improved classical CD4^+^ T cell help, and a cognate mechanism involving direct interactions with B cells via agonists presented on CD1d. For non-cognate help, both the α-GalCer and antigen must first be acquired by DCs to facilitate the adjuvant activity of NKT cells to drive an antigen-specific CD4^+^ T cell response. These T cells can differentiate into follicular helper CD4^+^ T (T_FH_) cells that enter the B cell zone of lymphoid tissues to interact with B cells expressing B cell receptors (BCRs) specific for the native antigen; this interaction is facilitated by the capacity of B cells to internalize the protein, and process it to be presented via MHC Class II. The signals delivered in this classical T cell help interaction ultimately lead to germinal centre formation, immunoglobulin class switching, memory B cell differentiation and establishment of long-lived plasma cells that produce antibody [145, 146]. Support for such a non-cognate role for NKT cells comes from studies showing that α-GalCer can help CD1d-deficient B cells, implying the adjuvant function can enhance T_FH_ formation, or that NKT cell-derived soluble factors support B cell stimulation [147]. However, it has been shown that a proportion of NKT cells can adopt a T_FH_-like phenotype, referred to as NKT_FH_ cells. These cells exhibit upregulated expression of the transcription factor Bcl6, which is also necessary for the differentiation of T_FH_ cells, and secrete the T_FH_-associated cytokine IL-21 [148, 149]. Like T_FH_, they express PD-1, and also express CXCR5, a chemokine receptor that facilitates entry into the B cell zone. It is therefore unsurprising that NKT_FH_ have been shown to provide direct help to B cells presenting agonists via CD1d. In fact, protein vaccination with α-GalCer can elicit IgG responses in MHC Class II-deficient mice, demonstrating that NKT_FH_ cells can substitute for T_FH_ help to B cells [150]. Although CD40 signalling is regarded as the primary helper signal [147], a report surprisingly showed enhanced antibody responses using α-GalCer in mice with CD40L-deficient NKT cells [151]. While it remains possible that other helper factors such as ICOS are involved [152], it is also possible that NKT_FH_ help is qualitatively different to classical T_FH_ help to B cells. Certainly, the outcome from the two classes of follicular helper cells can be different. Indeed, IgG responses induced with protein antigens and α-GalCer in mice lacking CD4^+^ T cells are of shorter duration [49], and responses induced with NKT_FH_ cognate help alone can actually increase populations of T follicular regulatory (T_FR_) cells, a subset that inhibits germinal centre responses resulting in short-lived, low-to-medium affinity antibody responses [153]. Thus, while it is true that NKT cells can serve as universal helpers to initiate antibody responses without the need for considering epitopes for conventional help, the quality of responses can be compromised, which must be considered in vaccine design.

Overall, the requirement for NKT_FH_ or T_FH_ cells, or their coordinated dual activity, is likely to depend on the particular antigen used, and its formulation characteristics. In studies where α-GalCer was linked to a bacterial polysaccharide derived from *S. pneumoniae*, it was shown that direct help from NKT_FH_ cells, in the absence of antigen for conventional T_FH_ cells, was sufficient for the production of long-lived protective antibody responses [126, 154]. In contrast, responses did not persist, and long-lived memory B cell populations were not induced, when α-GalCer was conjugated to the small hapten molecule NP [148]. The different outcome from these studies is likely due to the complexity of the antigen attached, with the polysaccharide containing repetitive motifs that were more likely to engage multiple BCRs on the surface. Thus, in the absence of conventional T_FH_ cells, NKT_FH_ cells can provide a surrogate role, but only when some BCR cross-linking occurs. Notably, cognate help was only possible when the B cell antigen was directly conjugated to α-GalCer, allowing simultaneous B cell-uptake of both components via the BCR. In recent examples that likely exploit this mechanism, conjugate vaccines incorporating the tumour-associated carbohydrate antigens, MUC-1 [155], GM3 [156] or sialyl-TN (STN) [157] successfully induced antigen-specific IgG antibodies that were shown to induce antigen-specific tumour cell death through complement-dependent cytotoxicity. Interestingly, the GM3 antigen has been shown to suppress the IL-4 but not IFN-γ response of NKT cells to α-GalCer by inducing changes to the APC [158], suggesting the selection of antigen may have a significant effect on the phenotype of NKT cell help and consequently the antibody response. In further developments, a recent study evaluated a conjugate of α-GalCer formulated into liposomes to achieve multivalent display to help drive antibody responses to an attached carbohydrate antigen [105]. Interestingly, they found that the larger structures (∼400 nm) induced a bias towards IgG2a production over IgG1 when compared with the smaller structures (∼200 nm), suggesting size had an impact on polarization of the response. Although the mechanism is unclear, it may reflect delivery to functionally different APCs, suggesting there is a room for further evaluation of formulation of conjugates to improve specific outcomes.

As observed with vaccines for cellular immunity, encapsulating antigen and agonists within nanovectors for stimulating humoral immunity provides qualitatively superior activation of NKT cells than soluble α-GalCer, without inducing anergy. This generally translates to greater B cell activation, with higher-titre antigen-specific IgM and IgG responses that are maintained long term (for a recent example, see Shute *et al.* [121]). The level of antigen displayed, and capacity to induce BCR crosslinking are also likely to be factors that contribute to such responses. However, while such vaccines may be effective, this does not necessarily mean they can be combined with other current vaccines in human use, even if the extra NKT cell-mediated adjuvant activity might at first seem complementary. In a cautionary tale, in the study of Shute *et al.*, a PLGA-based nanovaccine containing α-GalCer and polysaccharide antigens was shown to induce IgM and IgG humoral memory and protection against *S. pneumoniae*, but failed to improve responses when combined with a current T-dependent vaccine, and actually inhibited responses by a current T-independent vaccine [121]. Thus, there are circumstances where NKT cells negatively regulate B cells, perhaps through a cytotoxic function. While this has been observed in the context of chronic inflammation [159], more studies are required to determine when this might be a concern in vaccination strategies.

## INDUCING TISSUE-RESIDENT T CELL RESPONSES

While we have focused on NKT cells in the primary lymphoid organs as the source of licencing signals by NKT cells, it is worth noting that NKT cells are distributed in other tissues, notably mucosal tissues and the liver. Depending on the route of administration, some of these populations will be activated by vaccines incorporating NKT cell agonists. Studies in mice showed that α-GalCer-adjuvanted vaccines could be successfully delivered via the mucosal routes (intranasal or gastric gavage) [47]. Although T cell priming may still have involved the draining lymphoid tissues, it is possible local activation of NKT cells at the mucosal sites facilitated T cell recirculation to these tissues. In a more recent study with conjugates incorporating malaria antigens, we reported an accumulation of resident memory T cells (T_RM_) in the liver [135]. The recent discovery of T_RM_ in diverse tissues has altered our understanding of adaptive immunity, with the protection afforded by prior infection or vaccination shown in many cases to be mediated by tissue-adapted T_RM_ that do not recirculate throughout the body. Reviewed elsewhere [160], T_RM_ exhibit key phenotypic, transcriptional and functional features that were initially established in mouse models of infection, and have since been translated to humans by tissue sampling approaches. Studies conducted in mice had shown that current malaria vaccines, based on injection of irradiated sporozoites, induce liver T_RM_, and that protection is lost when the induced T_RM_ are specifically deleted [161]. In line with this observation, our conjugate vaccines provided protection in malaria challenge models that correlated with the size of the T_RM_ population induced [135]. Accumulation of liver T_RM_ can be enhanced by local antigen presentation [161–163] or inflammation [161, 164], and the conjugates may provide both these stimuli by sustaining local peptide presentation and activating local NKT cells to induce transient inflammation, providing local cues that specifically support T_RM_ accumulation. We speculate that T cell priming occurs outside of the liver, involving NKT cell-licencing, and these T cells then seed liver T_RM_ accumulation. Such vaccines could find application in preventing or therapeutic treatment of other liver infections. In this context, it is worth noting that the control of hepatitis B virus is associated with the accumulation of cells that closely resemble T_RM_ [165], and strategies that facilitate virus-specific liver T_RM_ may ultimately control chronic disease. It is not yet known whether T_RM_ formation can be enhanced by NKT cell stimulation in other tissues where they are found in high numbers, but this is certainly worth investigating. Furthermore, although the role of T_RM_ in tumour immunology is still not well understood, intra-tumoural T cells expressing the integrin CD103, which is indicative of T_RM_, have been reported to correlate with a favourable prognosis in cancer patients (reviewed by Dumauthioz *et al.* [166]), and T_RM_ have been shown to have anti-tumour activity in preclinical models [167, 168]. It is therefore possible that activating tumour-resident NKT cells could improve T cell therapy by enhancing tumour-specific T_RM_ accumulation.

## CONCLUDING REMARKS AND PERSPECTIVES

**Figure 1:**
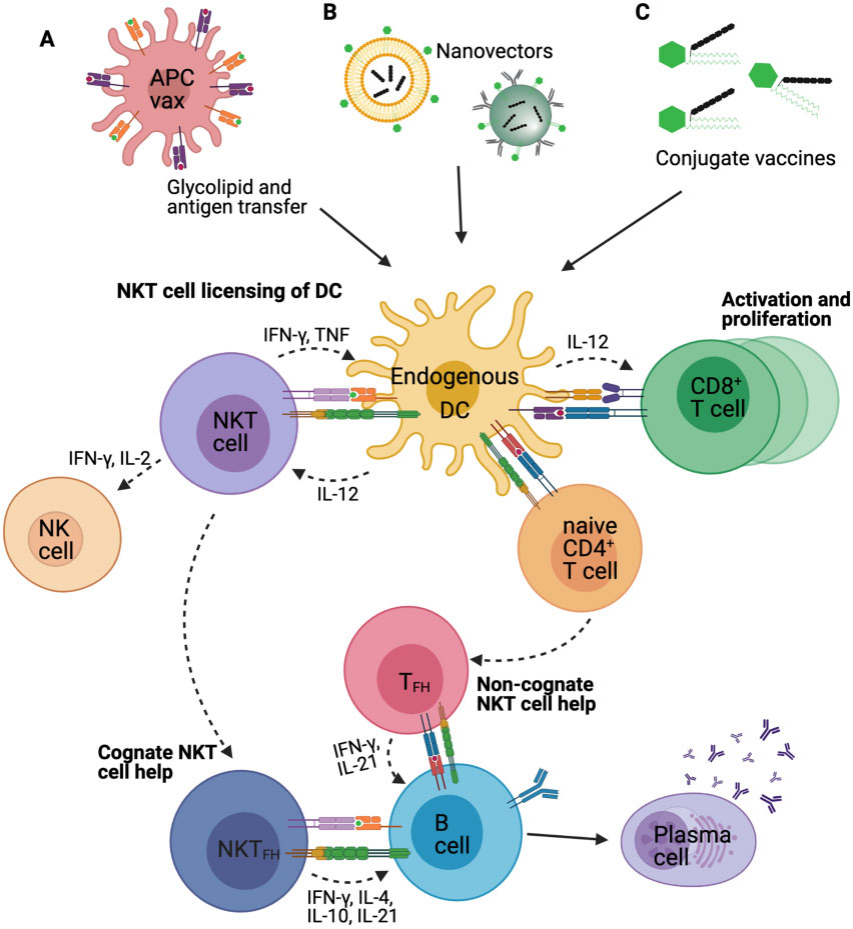
Strategies to focus NKT cell activity on APCs to improve vaccine outcome. (**A**) NKT cell agonists can be co-pulsed with antigen ex vivo onto vector cells such as monocytes, DCs, fibroblasts or even tumours cells before *in vivo* administration. Although injected APCs can theoretically be the recipients of NKT cell help, evidence suggests that the injected cells act as couriers to deliver the antigens and NKT cell agonists to DCs already resident in the host, which in turn receive licencing signals from NKT cells. (**B**) NKT cell agonists and antigen can be encapsulated into nanovectors, such as liposomes and nanoparticles that are either efficiently acquired by resident DCs, or express targeting moieties to aid this process. (**C**) Chemical conjugation of NKT cell agonists with antigen and glycolipid aids delivery to the same cell. By separating the components with a cleavage site for enzymes enriched in endogenous APCs, presentation of NKT cell agonist and antigenic peptide is enhanced by resident DCs. Licencing of resident DCs, involving CD40L/CD40 interactions, is particularly relevant to the cross-priming of CD8^+^ T cells, although it also facilitates proliferation and differentiation of CD4^+^ T cells. This can include differentiation into T_FH_ cells that provide help to B cells, promoting antibody class switching and stimulating their differentiation into long-lived antibody-producing plasma cells. Alternatively, activated NKT cells can differentiate into NKT_FH_ to provide help to B cells in the absence of conventional T_FH_. In either scenario, stimulation of antibody production is dependent on uptake of antigen by B cells via an antigen-specific BCR

The induction of CD4^+^ T cell help by vaccination can be confounded by issues of MHC diversity and epitope choice. In contrast, NKT cells can be universally activated by defined glycolipids presented via CD1d to provide a universal form of T cell help. Because of their high numbers in lymphoid tissues relative to antigen-specific CD4^+^ T cells, NKT cells are poised to deliver this help efficiently, without need for additional signals through pattern recognition. Ultimately, NKT cell agonists must be co-presented with antigenic peptides on the same APC to facilitate the licencing activities that drive T cells responses, while B cell responses can be supported indirectly through T_FH_ formation, or directly via NKT_FH_ cells. A variety of strategies have been highlighted here to achieve this (Fig. 1), of which the most practical for clinical development may be incorporation of antigen and agonists into liposomes or nanovectors, chemically conjugating these components together, or inserting agonists into the nanoparticles used for mRNA (or DNA) vaccines. The strategies could in fact be combined, although some evidence indicate that incorporating conjugate vaccines into nanovectors, such as liposomes, loses the advantage of the conjugate vaccine [123, 156], suggesting it may be unnecessary.

Rollout of vaccination programmes has been a global health success story, saving millions of lives each year. However, there is a low public tolerance for adverse events, and safety thresholds are understandably high for approval of new vaccines, particularly those given to healthy individuals as prophylaxis against infection. It is true that the potential severity of some chronic conditions may justify considering lower acceptable safety thresholds for therapeutic vaccines; nonetheless, to progress NKT cells agonists as new adjuvants, safety has to be a primary concern. Based on current limited clinical data, the injection of soluble unmodified NKT agonists is unlikely to meet acceptable safety thresholds, but incorporation into vectors or chemical conjugation may overcome this barrier. Not only can these approaches improve cell targeting to optimize the cellular interactions that enhance adaptive responses (with less unnecessary and potentially detrimental off-target activity and anergy), they can also be dose-sparing, with a likely improvement in safety profile. In this context, it is notable that a recent report showed that α-GalCer embedded in PLGA nanoparticles induced adjuvant activity in mouse studies at doses 1000 times lower than typically used with soluble α-GalCer [121].

The lower frequencies of NKT cells in humans compared with the commonly used preclinical rodent models have been regarded as a potential barrier to clinical efficacy of NKT cell agonists as immune adjuvants. It is therefore encouraging that adjuvant activities have been seen in outbred pigs with variable NKT cell frequency, and also in the trial with ABX196 in humans. In both cases, frequency of NKT cells in the periphery did not predict outcome, suggesting that it is the frequency and functionality of NKT cells in lymphoid tissues that are more important. Strategies to pre-screen recipients based on peripheral NKT cell frequency to select only those with high blood frequency for NKT cell-based therapy may therefore be unwarranted. Ultimately, we will not be able to draw strong conclusions on suitability of NKT cell agonists for human use until more clinical trials have been conducted.
